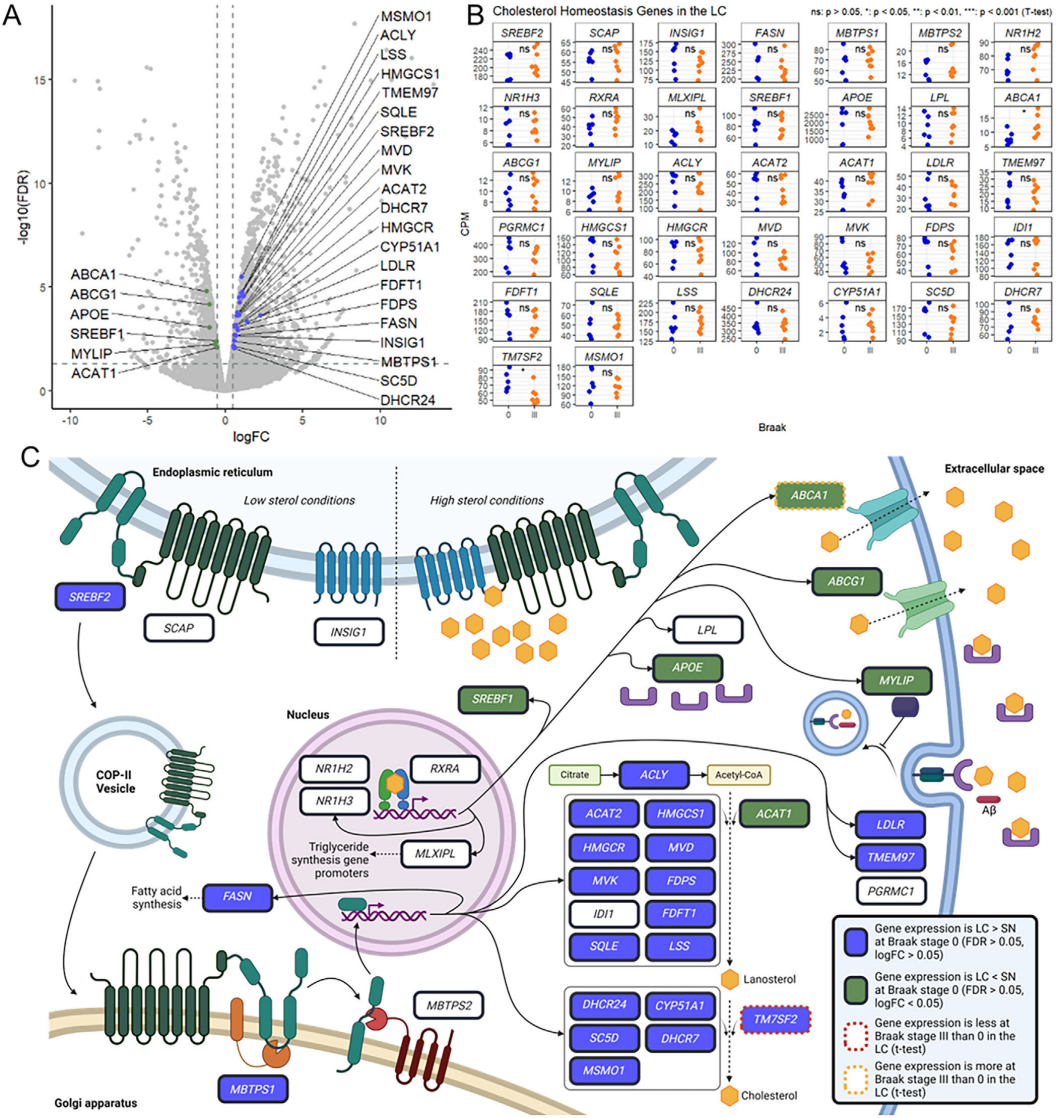# Pathways underlying selective vulnerability to Alzheimer’s disease in aminergic brainstem nuclei

**DOI:** 10.1002/alz.095326

**Published:** 2025-01-09

**Authors:** Alexander J. Ehrenberg, Cathrine Petersen, Felipe Luiz Pereira, Jessica J. Buxton, Claudia Kimie Suemoto, Renata Elaine Paraizo Leite, Roberta Diehl Rodriguez, Vitor Ribeiro Paes, Renata Eloah de Lucena Ferretti‐Rebustini, Eduardo Ferrioli, Ricardo Nitrini, Wilson Jacob‐Filho, Carlos Augusto Pasqualucci, William Seeley, Salvatore Spina, Daniela Kaufer, Lea T. Grinberg

**Affiliations:** ^1^ Memory and Aging Center, Weill Institute for Neurosciences, University of California, San Francisco, San Francisco, CA USA; ^2^ Helen Wills Neuroscience Institute, University of California, Berkeley, Berkeley, CA USA; ^3^ Innovative Genomics Institute, Berkeley, CA USA; ^4^ Weill Institute for Neurosciences and Memory and Aging Center, Department of Neurology, University of California, San Francisco, CA USA; ^5^ Gladstone Institute of Neurological Disease, San Francisco, CA USA; ^6^ Memory and Aging Center, UCSF Weill Institute for Neurosciences, University of California San Francisco, San Francisco, CA USA; ^7^ University of California, Berkeley, Berkeley, CA USA; ^8^ Division of Geriatrics, University of São Paulo Medical School, São Paulo, São Paulo Brazil; ^9^ Physiopathology in Aging Laboratory (LIM‐22), University of São Paulo Medical School, São Paulo, São Paulo Brazil; ^10^ Brazilian Brain Bank of the Aging Brain Study Group; University of São Paulo, São Paulo Brazil; ^11^ University of São Paulo Medical School, São Paulo, São Paulo Brazil; ^12^ Physiopathology in Aging Laboratory (LIM‐22), University of Sao Paulo Medical School, São Paulo, São Paulo Brazil; ^13^ Biobank for aging studies of the University of São Paulo, São Paulo Brazil; ^14^ University of São Paulo Medical School, São Paulo Brazil; ^15^ Hospital das Clínicas da Faculdade de Medicina da Universidade de São Paulo, São Paulo, São Paulo Brazil; ^16^ Division of Geriatrics, University of São Paulo Medical School, São Paulo Brazil; ^17^ Faculdade de Medicina da Universidade de São Paulo, São Paulo Brazil; ^18^ Physiopathology in Aging Laboratory (LIM‐22), University of São Paulo Medical School, São Paulo Brazil; ^19^ Memory & Aging Center, Department of Neurology, University of California in San Francisco, San Francisco, CA USA; ^20^ University of California San Francisco, San Francisco, CA USA; ^21^ Global Brain Health Institute, University of California San Francisco, San Francisco, CA USA; ^22^ Brain Bank of the Brazilian Brain Aging Study Group, São Paulo Brazil; ^23^ Memory and Aging Center, UCSF Weill Institute for Neurosciences, University of California, San Francisco, San Francisco, CA USA

## Abstract

**Background:**

The Neuromodulatory Subcortical System (NSS) consists of nuclei exhibiting early vulnerability to tauopathies, including Alzheimer’s Disease (AD). Within the NSS, there is a spectrum of vulnerability that becomes apparent in the earliest stages of AD, offering a chance to probe factors underlying vulnerability to AD.

**Method:**

In this study, we applied bulk RNA sequencing in well‐characterized postmortem human tissue from n = 22 cases at early (Braak 0‐III) AD‐tau stages to understand why this susceptibility gradient exists by examining two nuclei with very similar neurons but differing in their vulnerability to AD the locus coeruleus (LC) and substantia nigra (SN).

**Result:**

We identified 3421 differentially expressed genes between the LC and SN among Braak 0 cases. Gene set enrichment analysis directed attention towards cholesterol homeostasis, neuroinflammation, and oxidative stress as factors potentially underlying the differential vulnerability of the LC and SN to AD.

**Conclusion:**

Weighing the limitations of bulk RNA sequencing against the expression patterns of the constituitive genes within each gene set, we conclude that cholesterol homeostasis likely explains the differential vulnerability of the LC and SN to AD (Figure 1).